# Mechanical Properties and Hydration Degree of Magnesium Potassium Phosphate Cement Modified by Sintered Silt Ash

**DOI:** 10.3390/ma16217010

**Published:** 2023-11-02

**Authors:** Hongguang Zhang, Wenya Yang, Qiling Luo, Wu-Jian Long

**Affiliations:** 1 State Key Laboratory of Hydraulic Engineering Intelligent Construction and Operation, Tianjin University, Tianjin 300072, China; 2 Poly Changda Engineering Co., Ltd., Guangzhou 510620, China; 3 Guangdong Province Key Laboratory of Durability for Marine Civil Engineering, Shenzhen University, Shenzhen 518060, China

**Keywords:** magnesium potassium phosphate cement, mechanical properties, hydration degree, sintered silt ash, early-age cement

## Abstract

The effective utilization rate of river-dredged silt was extremely low, and common disposal methods such as dumping it into the ocean have already threatened the ecological environment. To demonstrate that dredged silt can be used as a mineral admixture to modify magnesium potassium phosphate cement (MKPC), the mechanical properties and hydration degree of sintered silt ash (SSA)-blended MKPC in the early stage of hydration were studied systematically in this paper, with MKPC as the reference group. The mechanical experiment results showed that in the process of increasing the SSA content to 25%, the compressive strength first increased and then decreased. Among the samples, the compressive strength of cement aged by 1d and 3d with 15% content was the highest, which increased by 11.5% and 17.2%, respectively, compared with the reference group. The setting time experiment found that with the increase in SSA content, the hydration reaction rate of MKPC slowed down significantly. Its effect of delaying hydration was most obvious when the SSA content was 10–15%. The X-ray diffraction pattern showed that there was no large amount of new crystalline substances formed in the hydration product. The results obtained by scanning electron microscopy show that the microstructure tended to be denser and the hydration products tended to be plump when the SSA content was in the range of 0–15%. The non-contact electrical resistivity experiment showed that the addition of SSA delayed the early hydration of MKPC. Combined with the above experiment results, it was found that when the content of SSA was less than 15%, it not only delayed the early hydration of MKPC, but also deepened its hydration degree.

## 1. Introduction

Magnesium phosphate cement (MPC) has excellent properties such as rapid hardening, early strength, and good adhesion. It is used widely in bioceramics [1,2,3,4], engineering quick fixes [5,6,7], solidification of harmful substances [8,9], protective coatings [10,11], and in the 3D printing [12] field. MPC is mainly composed of dead burnt MgO and soluble phosphate. The main strength is provided by struvite formed in the vigorous acid–base neutralization reaction [13]. The phosphates used commonly in MPC are NH_4_H_2_PO_4_ and KH_2_PO_4_. Research shows that NH_4_H_2_PO_4_ released ammonia during the hydration process and caused environmental pollution, so KH_2_PO_4_ is more suitable as a raw material for MPC. When KH_2_PO_4_ is used as the raw material of MPC, the cement is called magnesium potassium phosphate cement (MKPC). Although MPC has many advantages, its rapid hardening nature makes it unable to achieve sufficient hydration, and reduces its convenience in construction operations. Researchers have added different admixtures to MPC to improve its performance. Admixtures used commonly were mineral admixtures (fly ash [14,15,16], silica fume [14,15,17,18], metakaolin [19,20,21,22], slag [23,24,25]), fiber material [26,27,28,29], and polymer materials [3,30]. Other researchers conducted thermodynamic simulation studies on MPC [31]. In addition, algorithms were often used in research in the field of materials science [32,33,34,35].

At present, there are two main views on the hydration mechanism of MKPC, which are the local chemical reaction mechanism [36] and the through-solution mechanism. The local chemical reaction mechanism states that the crystallization of magnesium ammonium phosphate is a local chemical reaction that controls the hydration reaction. After forming on the surface of magnesium oxide, hydration products invade the interior magnesium oxide, and magnesium oxide continues to dissolve, causing the pH value to rise. When the pH > 7, magnesium ammonium phosphate crystals are produced and form a network structure. However, the through-solution mechanism is more accepted by people, which is divided into the following stages [37]: First, KH_2_PO_4_ dissolves in water and releases H^+^ to make the solution slightly acidic. MgO dissolves under this condition and releases Mg^2+^ gradually. Second, Mg^2+^ reacts with water molecules in the aqueous solution to form hydrated sol Mg(H_2_O)_6_^2+^, then these hydrated sols react with PO_4_^3−^, HPO_4_^2−^, and H_2_PO_4_^−^ in the solution to generate various hydration products. Third, as the polymerization reaction continues, the sols continue to cement to form a gel and further form a grid structure. Fourth, the grid gel gradually becomes saturated and transforms into the crystalline hydration product K-struvite. It covers the surface of incompletely reacted MgO particles and forms a structure with MgO particles as the skeleton and K-struvite as the cementing material.

More than 600 million cubic meters of river-dredged silt are produced globally every year, but less than 1% of it has been recycled, and most of it was dumped into the ocean for disposal. In 2015, nearly 140 million cubic meters of dredged silt from rivers were dumped into the sea in China [38]. The waste silt produced by the dredging of rivers and lakes has already threatened the ecological environment. How to treat it harmlessly is an urgent problem to be solved. The dredged silt produced in different environments has different components, but the main components are still Si, Al, Fe, other oxides, and humus. The existence of mineral components in dredged silt makes it possible to use it in inorganic cementitious materials. At present, many scholars have conducted systematic research on the modification of Portland cement with silt [39,40,41,42,43,44]; however, there have been few studies on the modified MPC of dredged silt.

Therefore, this paper studied the influence of dredged silt on the mechanical properties and hydration process of MKPC in the early stage of hydration through experiments. The mechanical properties were studied using compressive strength experiments. Setting time experiments characterized the effect of dredged silt on changes in the hydration rate of MKPC. The resulting crystalline hydration products were analyzed using X-ray diffraction (XRD), then the microstructure and hydration products were examined with scanning electron microscopy (SEM) and energy dispersive spectrometry (EDS). The change in resistivity during the hydration process was characterized with the non-contact electrical resistivity method (NC-ERM), and the influence of dredged silt on the hydration process of MKPC was evaluated further. The experimental results of compressive strength show that 15% SSA was better than 0%, 5%, 10%, 20%, and 25%. It was believed that the hydration product structure of 15% SSA was arranged so that it has better strength at the same age. At the same time, the development of hydration products was fuller and the degree of hydration was deeper. Comparison of experimental results using the above three mix ratios was accurate and meaningful for this study. Therefore, 0%, 15%, and 25% SSA were selected for experiments and comparative analysis in the XRD, SEM, and NC-ERM experiments.

## 2. Materials and Methods

### 2.1. Raw Materials

MKPC is composed of dead burnt MgO and KH_2_PO_4_. The MgO used was produced in Liaoning, which was made of magnesite calcined at 1500–1700 °C and then ground. Table 1 shows its chemical composition with a purity of 92%. The analytical pure KH_2_PO_4_ was obtained from Sinopharm Chemical Reagent Co., Ltd. (Shanghai, China), with a purity greater than 99.5%, a solubility of 22.6 g/100 mL at 20 °C, and is in line with the technical requirements for KH_2_PO_4_ in GB/T 1274-2011. Untreated silt was produced from Baiyangdian Hebei. Its fluidity was poor and its water content was too high, so it needed to be treated before being used. The treatment process is introduced in detail later. The chemical composition of the treated silt is shown in Table 2, and it can be seen that it had a high content of SiO_2_ and Al_2_O_3_. The water used was municipal tap water. In the study, different proportions of sintered silt ash (SSA) were used to replace part of the MgO, while keeping the mass ratio of KH_2_PO_4_/(MgO + SSA) at 0.4. The water/binder ratio was 0.24 uniformly. In the early stage of the experiments for this thesis, a literature review and many pre-experiments were conducted. KH_2_PO_4_/(MgO + SSA) = 0.4 and water/binder = 0.24 enabled the specimen to perform better. The mix design is shown in Table 3.

### 2.2. Treatment Process of Dredged Silt

**Figure 1:**
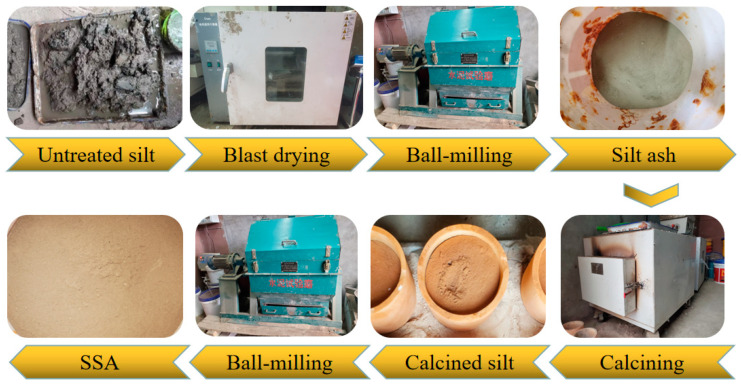
Treatment process of dredged silt.

Untreated silt had a high water content and poor fluidity, and needed to be treated before being used in experiments. The process of silt treatment was divided into four steps as shown in Figure 1: First, untreated silt was placed in a drying oven and dried to constant weight at 105 °C. Second, the dried silt was ball-milled for 10 min until the sludge became ash. Third, the sludge ash was continuously calcined in a high temperature box at 800 °C for 2 h. Fourth, the silt ash calcined at high temperature was ball-milled again for 10 min. This was due to the agglomeration of the silt ash calcined at high temperature.

### 2.3. Specimen Preparation

Unlike Portland cement, MKPC has the properties of quick hardening and early strength. Therefore, the ages of 1d and 3d of MKPC were selected to study its early hydration. KH_2_PO_4_ was granular crystals, and it was pulverized into powder by using a grinder to facilitate sufficient hydration. MgO, KH_2_PO_4_, and SSA with masses corresponding to the designed mix ratio were put into the mixing pot. The dry material was evenly mixed under the fan blade rotating at low speed. The corresponding weight of water was poured into the cement mixer and stirred at low speed for 60 s. The mixture was stopped stirring for 30 s. During this time, the remaining experimental material on the mixer blade was scraped into the pot and stirred at low speed for 30 s. For the experimental block used to test compressive strength, the mixed MKPC was poured into a 40 mm × 40 mm × 40 mm mold. Then, it was vibrated on a vibrating table for 30 s, and the excess MKPC on the surface was scraped off and covered with plastic film. After 1 h, the mold was removed and the test block was put into the standard curing room for curing for 1d and 3d. For NC-ERM experiment specimens, the prepared MKPC was poured into a specific ring-shaped experimental platform. The mold containing MKPC was held in both hands in a support position and rotated rapidly. This rotation was repeated several times clockwise and counterclockwise in the horizontal direction to make the experimental sample surface flat and highly consistent. The sides of the sample mold were lightly tapped with fingers to release the air in the MKPC.

### 2.4. Experiment Methods

#### 2.4.1. Compressive Strength

In the experiment, cubic experimental blocks with a size of 40 mm × 40 mm × 40 mm were used to test the compressive strength. The arithmetic mean of six experimental blocks in each group was used as the compressive strength. If one of the six measured values exceeded 10% of the arithmetic mean, this value was removed and the arithmetic mean of the other five values was taken. The specimen ages were 1d and 3d. The test instrument used was a YAW-300C automatic pressure testing machine, which was loaded at a constant speed of 2.4 kN/s until the sample broke. The experiment complied with the relevant regulations in GB/T 17671-2011.

#### 2.4.2. Setting Time

According to the design mix ratio in Table 3, the setting time of MKPC was measured using a Vicat apparatus (Jiangsu, China). The test needle was selected as the sample stick, and the pointer was aligned with the zero point of the scale when the test needle touched the glass plate. The slurry prepared according to the proportions was immediately loaded into the round mold and vibrated at once. The time point at which water was added and mixed was used as the timing starting point. The test needle was kept in contact with the slurry surface, and then the tightened screw was suddenly loosened after 1–2 s. When the test needle sank to 4 ± 1 mm from the bottom, it reached the initial setting state. At this point, the timer was stopped and recorded as the initial setting time. Measurements were taken every 10 s. When the initial setting time was approaching, 10 s was changed to 5 s. According to the research by Wang Erqiang et al. [45], when 0.5% boric acid was added to MKPC as a retarder, the initial and final setting times were 432 s and 518 s, respectively. The difference between them was only 86 s. Given the very short time interval, the initial setting time was taken as its setting time.

#### 2.4.3. XRD

When monochromatic X-rays irradiated the crystal, the atoms inside the crystal were arranged in a regular order to form a unit cell structure. The distance between these atoms was on the order of the wavelength of the incident X-rays. Therefore, the scattering of X-rays by different atoms interfered with each other, and strong X-ray diffraction appeared in some specific directions. The distribution and intensity of diffraction lines in space were closely related to the crystal structure. The diffraction pattern presented by each crystal reflected the arrangement of atoms inside the crystal. Consequently, the composition of the specimen could be characterized by the diffraction pattern obtained from the XRD experiment.

Specimens for XRD were taken from the core of the broken experimental blocks after the compressive strength experiment. The specimen was soaked in isopropyl alcohol for 48 h, and the free water in the open pores was displaced. Then, they were dried in a vacuum oven for 48 h. The purpose of above operation was to terminate the continuous hydration of the specimen, so as to obtain the specimen that was closest to the hydration state of the corresponding age. The vacuum-dried block specimens were fully ground into powder. The average particle size of the powder was controlled within 10 μm. There was no obvious graininess when touching the powder with hands. An appropriate amount of grinding powder was filled into the grooves of the sample holder, and the sample was compacted with a glass slide. Excess powder was removed and the sample surface was recompacted to be flush with the edge of the sample holder. The sample stage was inserted into the XRD diffractometer sample stage and the protective cover was closed. The instrument model used for XRD was the D8 ADVANCE, Bruker, Germany. The scanning angle was 10°–80°, and the speed was 10°/min.

#### 2.4.4. SEM and EDS

SEM uses a high-energy, highly focused electron beam to scan the surface of the specimen, and stimulate various physical information through the interaction between the electron beam and the substances in the specimen. Then, this information is collected, amplified, and re-imaged to achieve the goal of characterizing and studying the microscopic morphology of matter. When electrons interact with matter, the focused incident electrons excite primary X-rays. The characteristic X-rays emitted by different elements have different wavelengths and different energies. EDS is based on this principle to detect elements present in a specimen.

The specimens for SEM and EDS detection were still taken from the core of the broken experimental block after the compressive strength experiment. Unlike the XRD experiment, the block specimen taken needed to be as flat as possible for easy observation. The subsequent steps of soaking the sample in isopropanol and drying in a vacuum oven were consistent with the preparation of XRD specimens. The surface of the specimen was sprayed with gold before observation to enhance the conductivity of the specimen. The instrument model used for SEM was the Tescan Vega3, Brno, Czech Republic.

#### 2.4.5. NC-ERM

**Figure 2:**
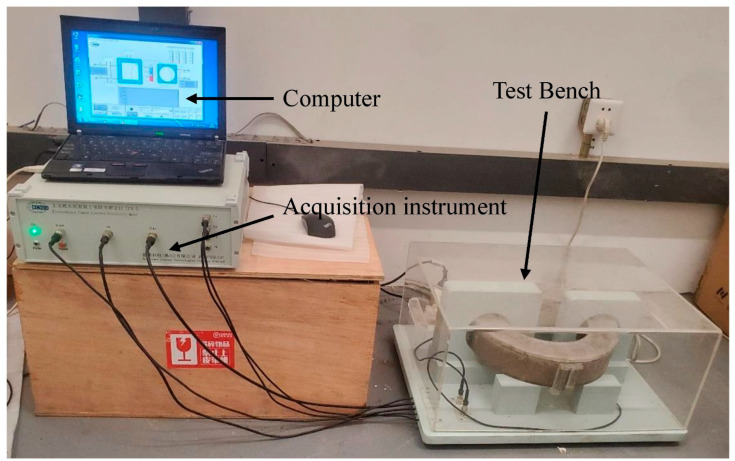
CCR-5.

There were contact problems between the electrode and the specimen to be tested when using the traditional resistivity measurement method [46], such as cracking and polarization effects. NC-ERM measured the resistivity of the specimen in a non-contact manner, so it had high precision. The experimental equipment used was the Electrodeless Cement Concrete Resistivity Meter (CCR-5, China), as shown in Figure 2. It consists of three parts: acquisition instrument, test bench, and computer. After the specimen was added to the specimen stage and vibrated, the computer and the acquisition instrument started to monitor and record the change in resistivity. Resistivity was recorded every minute for 72 h. After the recording was completed, the heights of 5 scattered points on the circular specimen were measured and recorded as h_1_–h_5_, and the arithmetic mean h of the 5 heights was taken as the average height. h was input into the CCR-5 system for height calibration to obtain accurate resistivity measurement data.

## 3. Results and Discussion

### 3.1. Mechanical Properties

**Figure 3:**
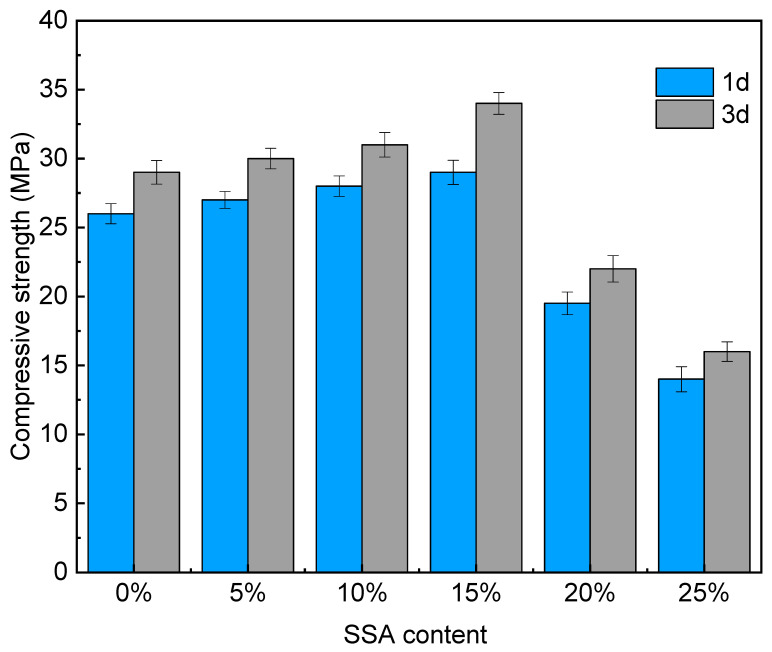
Compressive strength.

Figure 3 shows the compressive strength of MKPC at different contents of SSA and different ages. It was found that with the increase in SSA content, the compressive strength of 1d and 3d old MKPC both showed a trend of first increasing and then decreasing. When the SSA content was 15%, the compressive strength of the specimen reached the maximums of 29 MPa (1d) and 34 MPa (3d), which are increases of 11.5% and 17.2%, respectively, compared with that of MKOC with 0% SSA content. The reason might be that the incorporation of a small amount of SSA into MKPC could micro-fill the pores of the hydration product, which was beneficial to enhance the compressive strength. When the content of SSA was greater than 15%, the compressive strength decreased significantly. Compared with 0% SSA, the 1d compressive strength of 20% and 25% SSA decreased by 25% and 46.2%, respectively. The 3d compressive strength decreased by 24.2% and 44.8%, respectively. It was possible that a large amount of SSA replaced too much MgO, thereby reducing the formation of effective hydration products. In addition, SSA still contained many inert substances, and excessive addition will inevitably have an adverse effect on the macroscopic mechanical properties.

### 3.2. Setting Time

**Figure 4:**
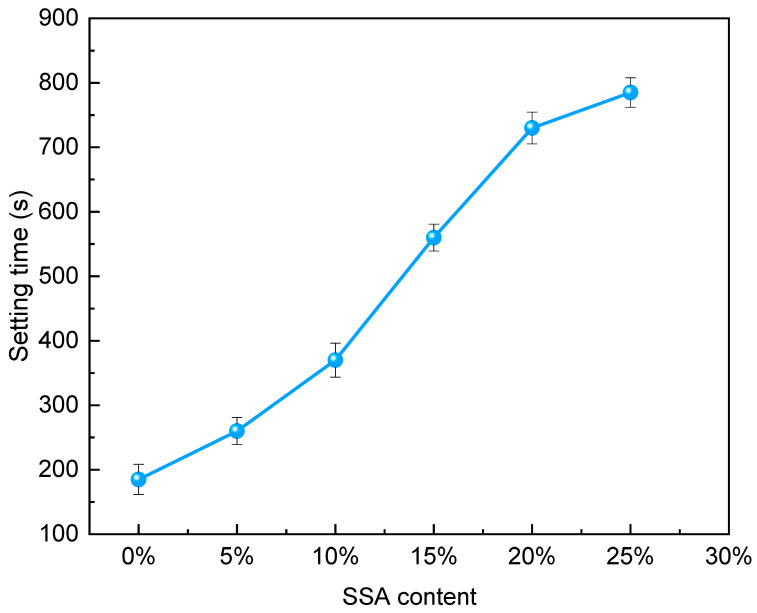
Setting time.

Figure 4 shows the setting time curve of MKPC with different SSA contents replacing MgO. The setting time showed an increasing trend with the increase in SSA content. Compared with 0% SSA, the growth rate of setting time of 5–25% SSA was 40.5%, 100%, 202.7%, 294.6%, and 324.3%, respectively. This means that the incorporation of SSA helps to slow down the reaction rate of MKPC. With the increase in SSA content, the growth rates of two adjacent SSA contents were 40.5%, 42.3%, 51.4%, 30.4%, and 7.5%, respectively. The growth rate of setting time showed a trend of first increasing and then decreasing, and the growth rate of the setting time was the largest in the transition through 10–15% SSA. SSA had a retarding effect on the setting time of MKPC, which may be due to the following two reasons. On the one hand, it might be due to SSA replacing some MgO, reducing the number of binding raw materials used. On the other hand, it might be because SSA wraps around the surface of MgO, temporarily reducing the effective contact area.

### 3.3. Hydration Products

**Figure 5:**
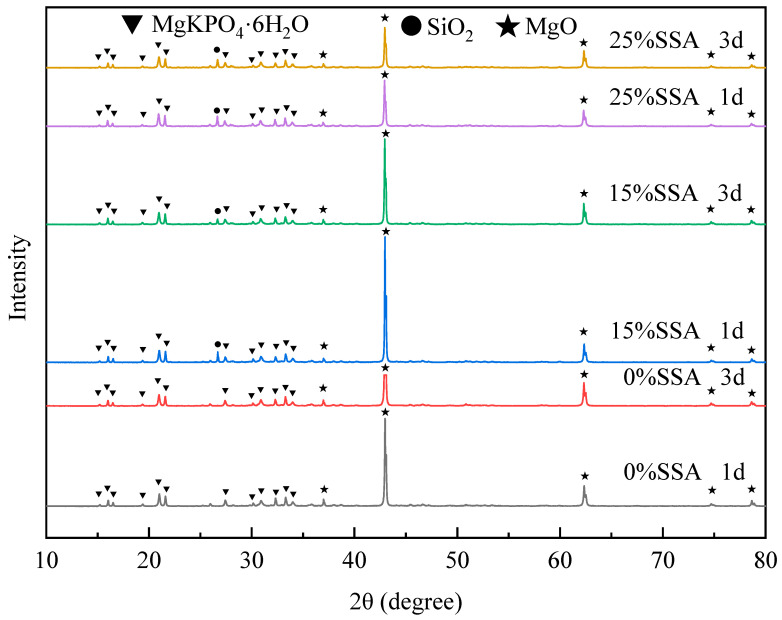
XRD pattern.

Figure 5 shows the XRD diffraction patterns of 1d and 3d old MKPC when the SSA contents were 0%, 15%, and 25%. There were sharp and strong SiO_2_ peaks in the 15% and 25% SSA spectra, but there was no obvious peak intensity at this position in 0% SSA, because SiO_2_ in MKPC was provided mainly by SSA. At the same SSA content, the peak intensity of SiO_2_ weakened with the increase in age. The reason may be that with the large amount of heat of hydration released during the hydration process, SiO_2_ was activated in an alkaline environment to form a Si-containing amorphous phase and was consumed [22,47]. These amorphous phase products could not show obvious peak intensities in XRD. MgO in the raw material was in excess relative to KH_2_PO_4_, and unreacted MgO showed a sharp peak intensity in the spectrum. The peak intensity of MgO in 15% SSA was significantly stronger than that in 0% and 25%, which may be because that SSA coated the surface of MgO and slowed down its nucleation. At the same SSA content, the peak intensity of MgO weakened with the increase in curing age, indicating that unreacted MgO was still slowly participating in hydration. Combined with the experiment results of compressive strength, 15% SSA had a temporary delay effect on hydration, but had a promotion effect on the degree of hydration. There were many peaks of MgKPO_4_·6H_2_O (K-struvite) in the range of 15°–35°, which was the main hydration product of MKPC. The broad hump-like peaks at 25°–40° represented the formation of a gel phase. One possible reason was that the raw material MgO and Al_2_O_3_ provided by SSA formed an amorphous phase of Al during the hydration process [22]; another aspect might be the aforementioned amorphous phase of Si. After the analysis of the peak type, no obvious new characteristic peaks appeared after the addition of SSA, which indicated that numerous new crystal-like substances were not formed in the hydration product.

### 3.4. Microscopic Morphology

**Figure 6:**
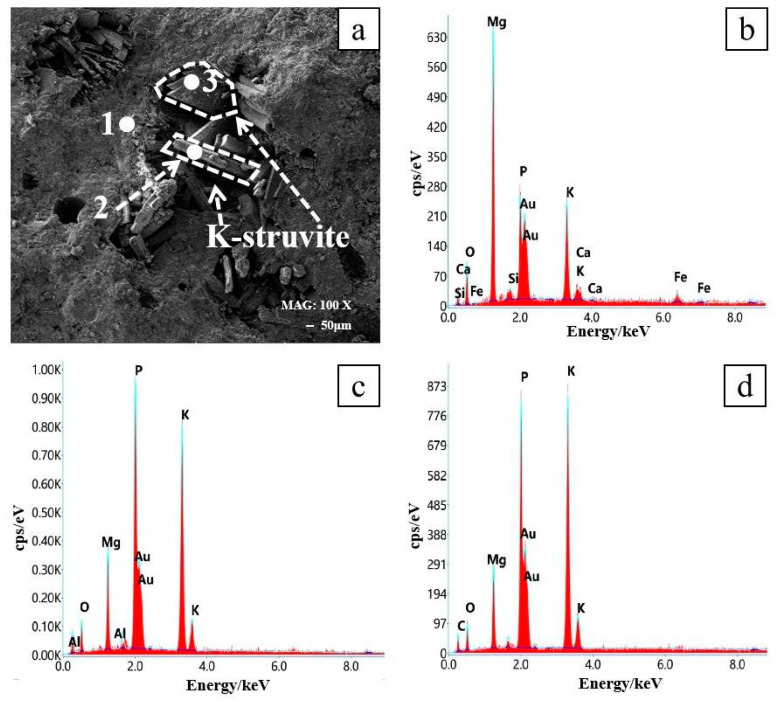
Content of chemical elements at three spots in Figure 6a. (**a**) Three spots; (**b**) EDS of spot 1; (**c**) EDS of spot 2; (**d**) EDS of spot 3.

Figure 6 shows three spots under SEM and their EDS results in the 0% SSA specimen. (a) shows the surface of the specimen aged 1d at 0% SSA at 100× magnification. It was observed that the surface structure of the specimen was weakly dense and there were many holes. Meanwhile, loose sheet-like [48,49] and rod-like [50,51] structures could be observed clearly. The EDS results in (c) and (d) show that spots 2 and 3 contained a large amount of P and K. Combined with the shape of the hydration product, it was determined that this was K-struvite. The EDS result of spot 1 in (b) shows a very significant Mg content, which was speculated to be unreacted MgO covering the K-struvite.

**Figure 7:**
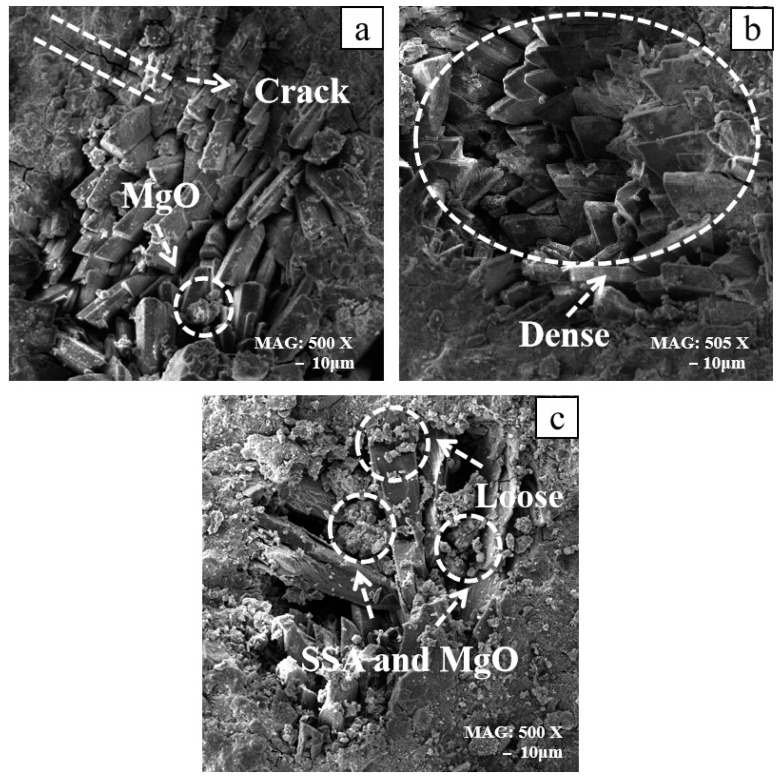
Microscopic morphology at 1d: (**a**) 0% SSA-500X, (**b**) 15% SSA-505X, and (**c**) 25% SSA-500X.

Figure 7 shows the electron microscopy images of 0%, 15%, and 25% SSA specimens at 1d of age. (a) shows the 500X magnified morphology of the MKPC specimen at the age of 1d at 0% SSA content. It can be observed that there were gaps between the stacked flaky K-struvite, and MgO that had not participated in the reaction was attached to the surface. Microcracks on the sample surface might be related to hydration shrinkage, which adversely affected the compressive strength. (b) shows the microscopic morphology of 15% SSA at 505X magnification at the age of 1d. Numerous plate-shaped and columnar K-struvite crystals were stacked, developed, and fully dense in structure. Compared with 0% SSA, the inert materials in SSA that did not participate in the reaction might have a filling effect on the intercrystalline pores of K-struvite. Combined with the results of the compressive strength experiment, this filling improved the macroscopic mechanical properties of MKPC significantly. (c) shows the microscopic morphology of 25% SSA at 500X magnification at the age of 1d. Compared with 15% SSA, the intercrystalline pores of K-struvite in 25% SSA were significantly larger, and a large amount of loose SSA was distributed on the surface. On the one hand, excessive SSA reduced the amount of MgO greatly in the raw material, thereby reducing the amount of K-struvite that played a major supporting role in the structural strength. On the other hand, it might be that the excessive SSA covered the surface of MgO, which seriously hindered the nucleation of MgO during the hydration process.

**Figure 8:**
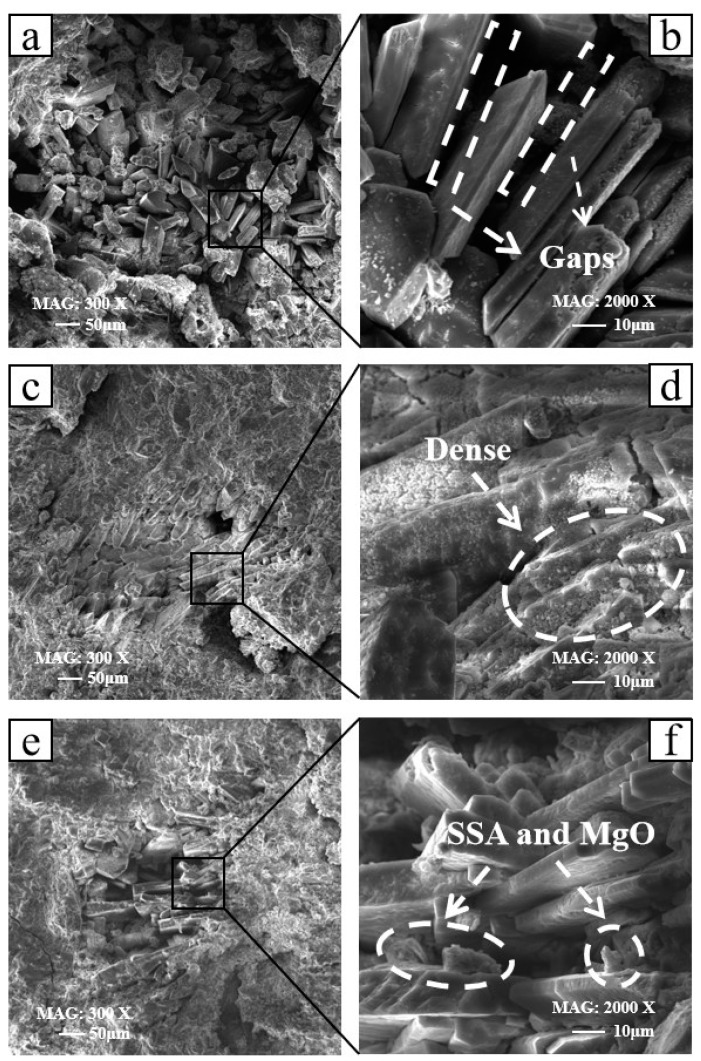
Microscopic morphology at 3d: (**a**) 0% SSA-300X, (**b**) 0% SSA-2000X, (**c**) 15% SSA-300X, (**d**) 15% SSA-2000X, (**e**) 25% SSA-300X, and (**f**) 25% SSA-2000X.

Figure 8 shows the microscopic morphology of 0% SSA, 15% SSA, and 25% SSA at 3 days of age under 300X and 2000X magnification. (b), (d), and (f) are partial enlarged images of (a), (c), and (e), respectively. In (a) and (b), it can be seen that the layered struvite was stacked and arranged, but the stack was loose and there were gaps. The layered struvite in (c) and (d) was closely stacked and had a dense structure. The layered struvite in (e) and (f) was mixed with loose MgO and SSA. Combined with the mechanical properties, this seriously shows the reduced mechanical performance of MKPC. By comparing the micromorphology of SSA with the same content in Figure 7 and Figure 8, it was found that the hydration products at the 3d age were plumper and denser than those at the 1d age. At the same time, there was less loose MgO and SSA attached to the hydration products at the 3d age.

### 3.5. Hydration Process

The conductivity of cement-based materials is derived from the ion transport largely in the pore solution, and the porosity and conductivity of the pore solution are important factors affecting the conductivity of cement-based materials [52]. The porosity can largely reflect the degree of hydration of cement, so the change in resistivity of cement-based materials during the hydration process can be used to characterize the degree of hydration.

**Figure 9:**
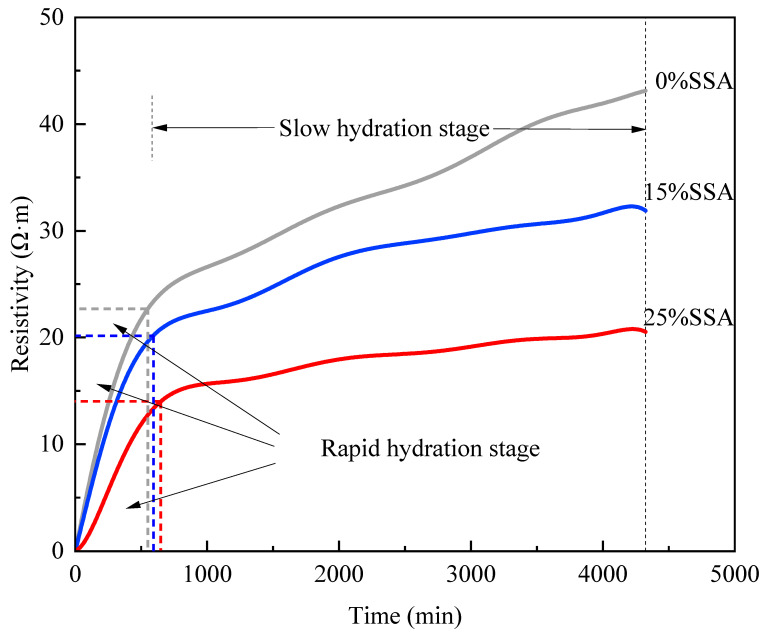
The resistivity development with time for MKPC during the first 72 h.

Figure 9 shows the curves of resistivity versus time for MKPC with SSA contents of 0%, 15%, and 25% within 0–72 h. The curves under the three SSA contents showed the same trend: they rose rapidly first and then rose slowly. According to the trend of the curve, the early hydration of MKPC could be divided into two stages: rapid hydration and slow hydration. During the rapid hydration stage, KH_2_PO_4_ dissolved H^+^ quickly in water, and underwent a violent acid–base neutralization reaction with Mg(OH)_2_ formed on the surface of MgO, which is slightly soluble in water, to form K-struvite and release a great amount of heat. The generated K-struvite accumulated gradually on the surface of MgO particles, which reduced the contact area between MgO particles and the outside world and delayed the hydration rate. At this time, it entered the slow hydration stage gradually. By comparing the curves of different contents of SSA in Figure 7, it could be found that with the increase in SSA content, the rising speed of the curve in the rapid hydration stage was slower, but the duration of this stage was gradually longer. Combined with the experimental results of the macroscopic mechanical properties of compressive strength, it was conjectured that the addition of 15% SSA covered the MgO particles partially to slow down the severe acid–base neutralization reaction at the beginning. Moreover, the stacking speed of K-struvite on the surface of MgO was lower than that of 0% SSA, which made more MgO participate in the reaction and generated more effective hydration products. For a suitable SSA content, the coating effect of SSA on MgO did not hinder its nucleation, but promoted the degree of hydration. In the slow hydration stage, the value of resistivity still maintained the same size relationship as that in the rapid hydration stage, with 0% SSA > 15% SSA > 25% SSA. The resistivity of MKPC with 25% SSA was significantly lower than that of 0% and 15% SSA within 72 h, mainly because a large amount of SSA replaced the raw material MgO, thereby reducing the formation of effective hydration products.

## 4. Conclusions

This paper systematically studied the influence of SSA on the mechanical properties and hydration process of MKPC in the early stage of hydration through experiments. The conclusions of this study are as follows:

(1) Under different SSA contents, the compressive strength of MKPC was significantly improved with a 15% content of SSA, which was 11.5% and 17.2% higher than 0% SSA at 1d and 3d ages, respectively. The 20% and 25% SSA content made the compressive strength of MKPC drop sharply. Compared with 0% SSA, the 1d compressive strength of 20% and 25% SSA content decreased by 25% and 46.2%, respectively. The 3d compressive strength decreased by 24.2% and 44.8%, respectively. SSA had an obvious delaying effect on the hydration rate of MKPC, and the delaying effect on the growth rate of setting time was the most obvious when the SSA content was 10–15%.
(2) Compared with 0% SSA MKPC, no obvious new crystal hydration products were detected in SSA-blended MKPC. When the SSA content was 15%, the micro-filling effect made the K-struvite crystal stack more compact.
(3) The addition of SSA delayed the early hydration process of MKPC. Compared with 0% SSA, 15% SSA content made the hydration rate of MKPC slower, but the hydration degree was deepened. The hydration degree of 25% SSA was lower than that of 0% SSA and 15% SSA.
(4) SSA can be used as a mineral admixture to modify MKPC. SSA-blended MKPC showed better mechanical properties and hydration degree at 15% SSA content.

## Figures and Tables

**Table 1:** Chemical composition of dead burnt MgO (%).

Composition	MgO	SiO_2_	CaO	Al_2_O_3_	Fe_2_O_3_	LOI
Content	91.04	4.73	2.09	0.86	0.94	1.01

**Table 2:** Chemical composition of treated silt (%).

Composition	SiO_2_	Al_2_O_3_	Fe_2_O_3_	CaO	MgO	SO_3_	f-CaO	LOI
Content	55.83	17.49	7.65	9.42	3.62	1.35	-	3.52

**Table 3:** Design of experiment mixing ratio (%).

MgO	SSA	KH_2_PO_4_/(MgO + SSA)	Water/Binder
100%	0%	0.4	0.24
95%	5%		
90%	10%		
85%	15%		
80%	20%		
75%	25%		

## Data Availability

Not applicable.
